# Leaner and greener analysis of cannabinoids

**DOI:** 10.1007/s00216-017-0256-3

**Published:** 2017-02-23

**Authors:** Elizabeth M. Mudge, Susan J. Murch, Paula N. Brown

**Affiliations:** 1 Natural Health & Food Products Research, British Columbia Institute of Technology, 3700 Willingdon Ave, Burnaby, British Columbia V5G 3H2 Canada; 2 Department of Chemistry, University of British Columbia, 3247 University Way, Kelowna, British Columbia V1V 1V7 Canada

**Keywords:** Green chemistry, Single-laboratory validation, *Cannabis*, Cannabinoids, Medical marijuana

## Abstract

There is an explosion in the number of labs analyzing cannabinoids in marijuana (*Cannabis sativa* L., Cannabaceae) but existing methods are inefficient, require expert analysts, and use large volumes of potentially environmentally damaging solvents. The objective of this work was to develop and validate an accurate method for analyzing cannabinoids in cannabis raw materials and finished products that is more efficient and uses fewer toxic solvents. An HPLC-DAD method was developed for eight cannabinoids in cannabis flowers and oils using a statistically guided optimization plan based on the principles of green chemistry. A single-laboratory validation determined the linearity, selectivity, accuracy, repeatability, intermediate precision, limit of detection, and limit of quantitation of the method. Amounts of individual cannabinoids above the limit of quantitation in the flowers ranged from 0.02 to 14.9% w/w, with repeatability ranging from 0.78 to 10.08% relative standard deviation. The intermediate precision determined using HorRat ratios ranged from 0.3 to 2.0. The LOQs for individual cannabinoids in flowers ranged from 0.02 to 0.17% w/w. This is a significant improvement over previous methods and is suitable for a wide range of applications including regulatory compliance, clinical studies, direct patient medical services, and commercial suppliers.

**Electronic supplementary material:**

The online version of this article (doi:10.1007/s00216-017-0256-3) contains supplementary material, which is available to authorized users.

## Introduction

**Fig. 1:**
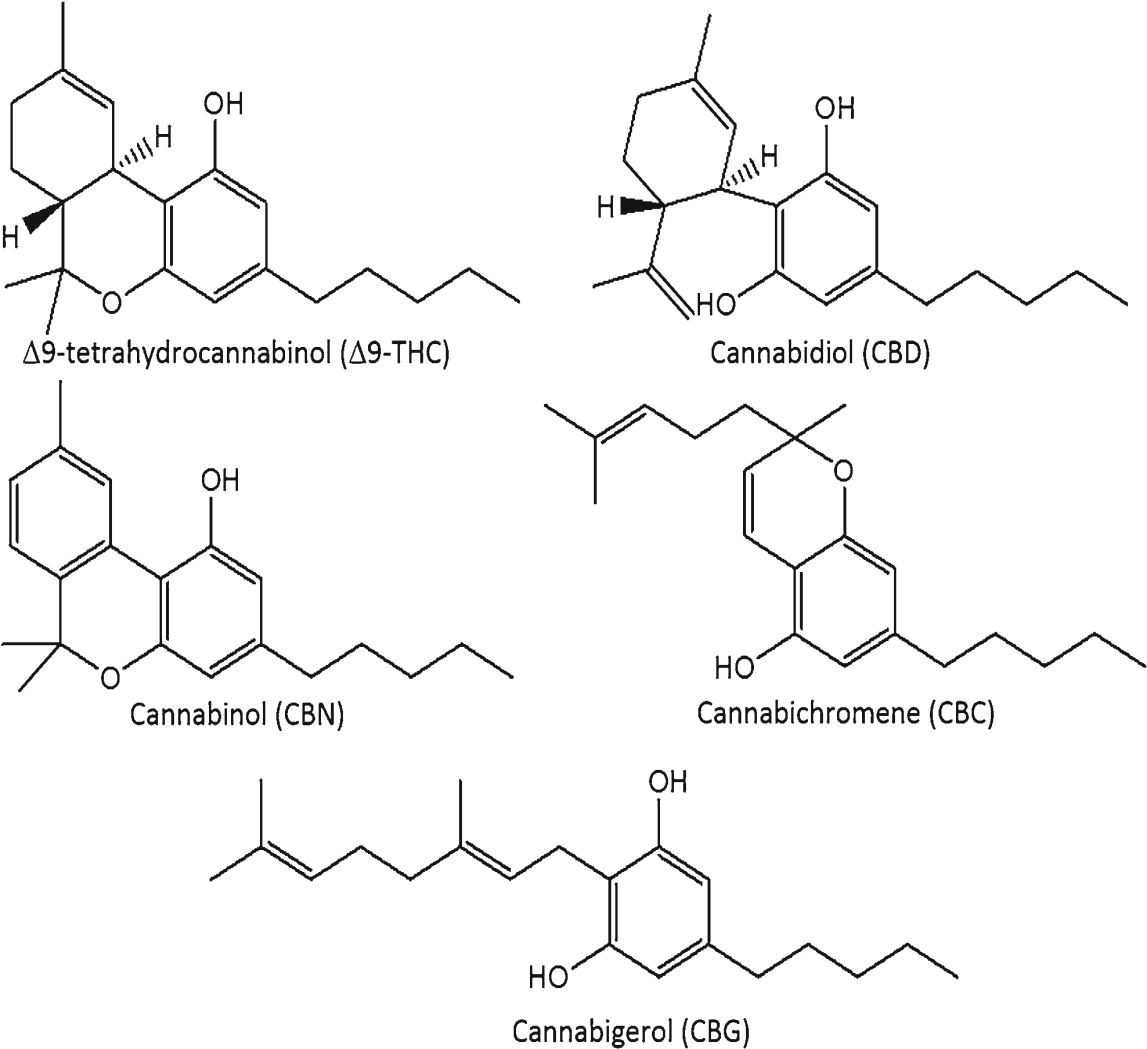
Structures for the main neutral cannabinoids found in *Cannabis* flowers

The modern cannabis market is in a period of dramatic flux. In the USA, cannabis is classified as a schedule I drug [1], but eight US states have legalized marijuana for recreational use and 28 states have allowed medical marijuana on the basis of evidence of anxiolytic, analgesic, sedative, anticancer, and appetite stimulation effects [2–5]. Regulations regarding *Cannabis* spp. vary globally. The Netherlands, Uruguay, and Portugal have decriminalized possession. In Canada, cannabis is a schedule II controlled substance, but regulations have allowed production for medical purposes through licensed producers and personal production licenses [6]. Canadian production of commercial products must take place in a facility using good manufacturing practices, and products must be assayed for the presence and quantity of Δ9-tetrahydrocannabinol (Δ9-THC), Δ9-tetrahydrocannabinolic acid (THCA), cannabidiol (CBD), and cannabidiolic acid (CBDA), using validated analytical methods [6]. In total, more than 100 cannabinoids in 11 subclasses have been characterized in cannabis and are concentrated in the glandular trichomes of the female inflorescences and other cannabinoids classes include cannabigerols (CBG), cannabichromenes (CBC), and cannabinols (CBN) (Fig. 1) [7]. The cannabinoids occur primarily in acid form, with neutral cannabinoids formed during drying, storage, and decarboxylation during smoking. Δ9-THC, the main psychoactive cannabinoid, can be over 20% by weight in specially bred cannabis strains [8, 9]. CBD, known for its anti-inflammatory activity and antagonism of Δ9-THC-induced anxiety, can range from below 0.5% up to 6.5% by weight [9, 10].

There are a significant number of analytical methods to quantify cannabinoids available, many of which do not provide sufficient validation data to establish the method performance and reliability. Without this information, there is a possibility that the methods are not fit for purpose. The solvent composition, mass to solvent ratio, extraction technique and time vary considerably between methods. Separations of cannabinoids use different mobile phases, columns and gradients, and given the number of minor cannabinoids present in authentic materials, there is a possibility for co-elution of peaks and inaccurate quantitative results [11, 12]. Rigorous validation procedures are necessary to ensure that the results of any analytical method are reliable. Without this data on method performance, the final method may not meet the needs of the users who adopt it for routine use, therefore producing inaccurate information pertaining to the products that people are using for the treatment of medical conditions [13]. The speed with which regulations have changed and the nature of the rapidly expanding cannabis marketplace have created increased pressure for fast, safe, simple, and accurate analysis of phytochemicals to meet the demands of high-throughput laboratories and rapid release of finished products.

The most commonly used extraction solvent for cannabinoid analysis is 9:1 methanol/chloroform (% v/v), with some exceptions [9, 11, 14–16]. It was originally selected to dissolve the internal standard di-*n*-octyl phthalate, which is no longer necessary with commercially available reference standards [16]. There is an increasing desire to find greener methods to reduce use of chlorinated solvents which can be toxic, expensive to dispose of, and hazardous to transport and store [17, 18]. Long-term, chronic exposure to chloroform is associated with liver and kidney damage, where the occupational exposure limit is 2 ppm in air [18, 19]. While laboratory safety procedures reduce exposure significantly, the risks of spills and inhalation of vapors are increased with chloroform use and there is a diversity of safety equipment used in the labs engaged in this analysis. Removal of chloroform from the extraction solvent will improve laboratory safety, reduce reagent and disposal costs, while improving the environmental impact associated with chlorinated solvent usage.

The objective of the current work was to develop a fully validated, simplified, green chemistry method for labs to implement that may not have high levels of expertise or capacity for method development or validation. We developed the method using statistically guided method development protocols for the quantitation of eight cannabinoids in *Cannabis* flowers and oils. Nine authentic *Cannabis* flower materials and one *Cannabis* oil with a wide range of cannabinoid contents were obtained and used as test articles for the validation of the method of the AOAC International guidelines [20]. This method does not use chlorinated solvents, reduces sample preparation time, and ensures precise and accurate determination of cannabinoids.

## Materials and methods

### Reagents

HPLC-grade methanol and acetonitrile were purchased from VWR International (Mississauga, ON, Canada). ACS-grade chloroform was obtained from VWR International. Water was purified to 18 MΩ using a Barnstead Smart2Pure nanopure system (Thermo Scientific, Waltham, MA). Ammonium formate for HPLC (>99.0%) was purchased from Sigma Aldrich (Oakville, ON, Canada) and formic acid was (98% pure) was purchased from Fisher Scientific (Ottawa, ON, Canada).

### Calibration standards

Certified reference materials (CRMs) were purchased from Cerilliant Corp (Round Rock, TX) for nine cannabinoids: Δ9-THC, THCA, Δ8-THC, CBD, CBDA, CBG, CBN, CBC, and tetrahydrocannabivarin (THCV). The individual cannabinoids were provided in solution at 1.0 mg/mL concentration certified by the supplier. The acidic cannabinoids were provided in acetonitrile and neutral cannabinoids in methanol. Fresh ampules were used for the validation study to ensure accurate quantitation of the individual constituents.

### Test materials

Dried medical marijuana samples were purchased from several licensed producers within Canada. Nine products were selected for a variety of cannabinoid concentrations ranging from 0.2% to 17% total THC and 0.3% to 9% total CBD. As a result of the legal restrictions pertaining to these products, voucher specimens were not possible, but were purchased directly from the source to ensure authenticity. A dried ethanol extract was dissolved in oil at a 1:10 dilution.

### HPLC analysis

An Agilent 1200 RRLC system equipped with a temperature-controlled autosampler, binary pump, and diode array detector (Agilent Technologies, Mississauga, ON, Canada) was used to separate the cannabinoids. The separation was achieved on a Kinetex® C18, 1.7 μm, 100 × 3.0 mm i.d. column (Phenomenex, Torrance, CA). Mobile phase compositions were (A) 10 mM ammonium formate, pH 3.6 and (B) acetonitrile using gradient conditions at 0.6 mL/min. The separation was achieved according to the following gradient: 0–8 min, 52–66%B; 8–8.5 min, 66–70%B; 8.5–13 min, 70–80%B; 13–15 min, 80%B. A 7-min column equilibration was performed after each run. The injection volume was 5 μL and detection was at 220 nm. The autosampler was maintained at 4 °C.

### Preparation of test materials

#### Plant tissues

A minimum of 5 g of dried flowers was ground together from each test sample to ensure sample homogeneity. Ground flowers were extracted by weighing 200.0 mg into a 50-mL amber centrifuge tube. Then 25.00 mL of 80% methanol was added and vortexed for 30 s. Extraction took place using a sonicating bath for 15 min where samples were vortexed every 5 min. Extracts were filtered with 0.22-μm Teflon filter, diluted either 1:2, 1:5, or 1:10 using the extraction solvent into amber HPLC vials, and stored at 4 °C until analysis.

#### Oil

Cannabis oil was mixed by inversion prior to sample preparation. Then 50.0 mg of oil was weighed into a 50-mL amber centrifuge tube to which 10.00 mL of methanol was added and vortexed for 30 s. Extracts were sonicated for 15 min with vortexing every 5 min. Samples were filtered with 0.22-μm Teflon filters into amber HPLC vials and stored at 4 °C until analysis.

### Method optimization

#### Analyte stability

Mixed calibration standards were stored at −20 °C, 4 °C, and 22 °C in the dark and tested at regular intervals to assess cannabinoid stability in solutions. Sample extracts were stored at 4 °C and 22 °C in light and dark conditions. A sample with greater than 5% loss from time zero was considered unstable.

#### Fractional factorial

The partial factorial design for method optimization and data analysis was completed using Minitab 16 (Minitab 16, State College, PA). Individual cannabinoids were quantified as percentage weight for weight in *Cannabis* flowers and milligrams per gram in oil. Microsoft Excel (Richmond, WA) was used for quantitative calculations and statistical analysis of validation data.

### Single-laboratory validation parameters

The optimized method was subjected to a single-laboratory validation according to AOAC International guidelines for dietary supplements [20]. Δ8-THC was not observed in any of the samples and therefore was not considered in the method validation.

#### Preparation of calibration solutions

Individual cannabinoid CRMs were used to prepare seven-point standard calibration curves for eight cannabinoids in concentrations ranging from 0.5 to 250 μg/mL. Dilutions of the CRMs were performed using the extraction solvent composed of 80% methanol. Concentration ranges were modified for each cannabinoid as summarized in Table 1. The calibration curves were plotted and the slope and *y* intercept for each cannabinoid were used for linear regression analysis. Calibration curves were visually inspected and correlation coefficients were determined. An *r* ^2^ of at least 0.995 was deemed suitable for quantitation. Mixed standards were stored at 4 °C and were stable for up to 3 days.

**Table 1:** Concentration of cannabinoids used in the calibration standards for the method validation and resolution of analytes in chromatographic separation

Cannabinoid	Approximate Concentration (μg mL^−1^)							Average correlation coefficients (*r* ^2^)	Resolution*
	Lin 1	Lin 2	Lin 3	Lin 4	Lin 5	Lin 6	Lin 7		
CBDA	250	200	100	50	25	10	5	0.9990	6.34
THCV	25	20	10	5	2.5	1	0.5	0.9993	1.75
CBD	50	40	20	10	5	2	1	0.9982	1.64
CBG	25	20	10	5	2.5	1	0.5	0.9995	1.85
CBN	25	20	10	5	2.5	1	0.5	0.9992	3.18
THCA	250	200	100	50	25	10	5	0.9992	1.95
THC	50	40	20	10	5	2	1	0.9983	2.89
CBC	25	20	10	5	2.5	1	0.5	0.9991	2.29

*Between component of interest and closest eluting peak

#### Selectivity

Selectivity was demonstrated by injecting the reference materials and raw flower extracts to evaluate the resolution between closely eluting peaks and potential interferences at 220 nm. Resolution of greater than 1.5 is deemed acceptable by AOAC guidelines [20]. Peak purity was verified for all cannabinoids of interest.

#### Repeatability and intermediate precision

Quadruplicate samples of each test material were prepared on a single day to evaluate the repeatability as relative standard deviation (% RSD) for the individual cannabinoids. Intermediate precision was determined by repeating the repeatability studies on three separate days. The within-day, between-day, and total standard deviations were calculated for each cannabinoid in each test material. HorRat values were calculated to assess the overall precision of the method [21].

#### Recovery

Recovery was determined at three concentration levels of the major cannabinoids: CBDA, CBD, THCA, and THC. Ground stinging nettle, used as the negative recovery material, was spiked with individual cannabinoids and prepared according to the sample preparation protocol.

#### Limits of detection and quantitation

The limits of detection and quantitation were determined using the US Environmental Protection Agency (EPA) method detection limit (MDL) protocol [22]. The MDL is defined as the minimum concentration of substance that can be measured and reported with 99% confidence that the analyte concentration is greater than zero. Extract solutions containing low concentrations of the cannabinoids were used to evaluate the method limits. Seven replicates were injected and the calculation for MDL was determined as the standard deviation of the calculated concentration between the seven replicates multiplied by the *t* statistic at 99% confidence interval. LOQ was determined as 10 times the standard deviation for the replicates to determine the MDL.

## Results

### Method optimization

A statistically guided optimization plan was developed using a partial factorial design to determine the impact of four parameters used in cannabinoid extractions from dried flowers.

#### Extraction of tissues

**Fig. 2:**
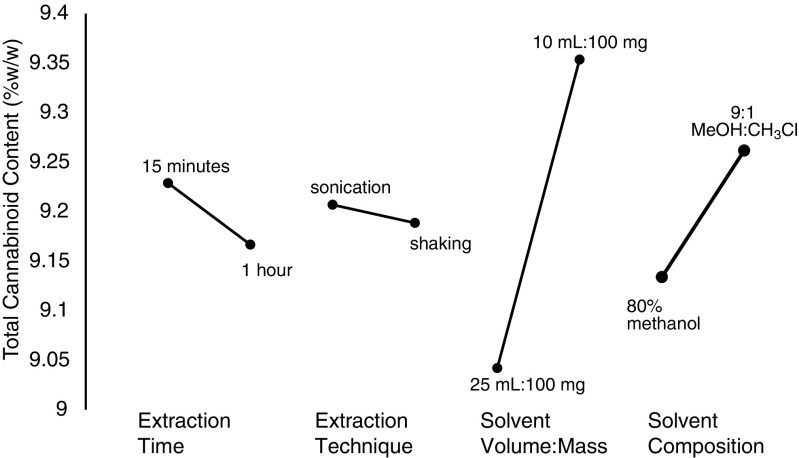
Variation of total cannabinoids determined using a two-level partial factorial design to optimize the sample preparation

**Fig. 3:**
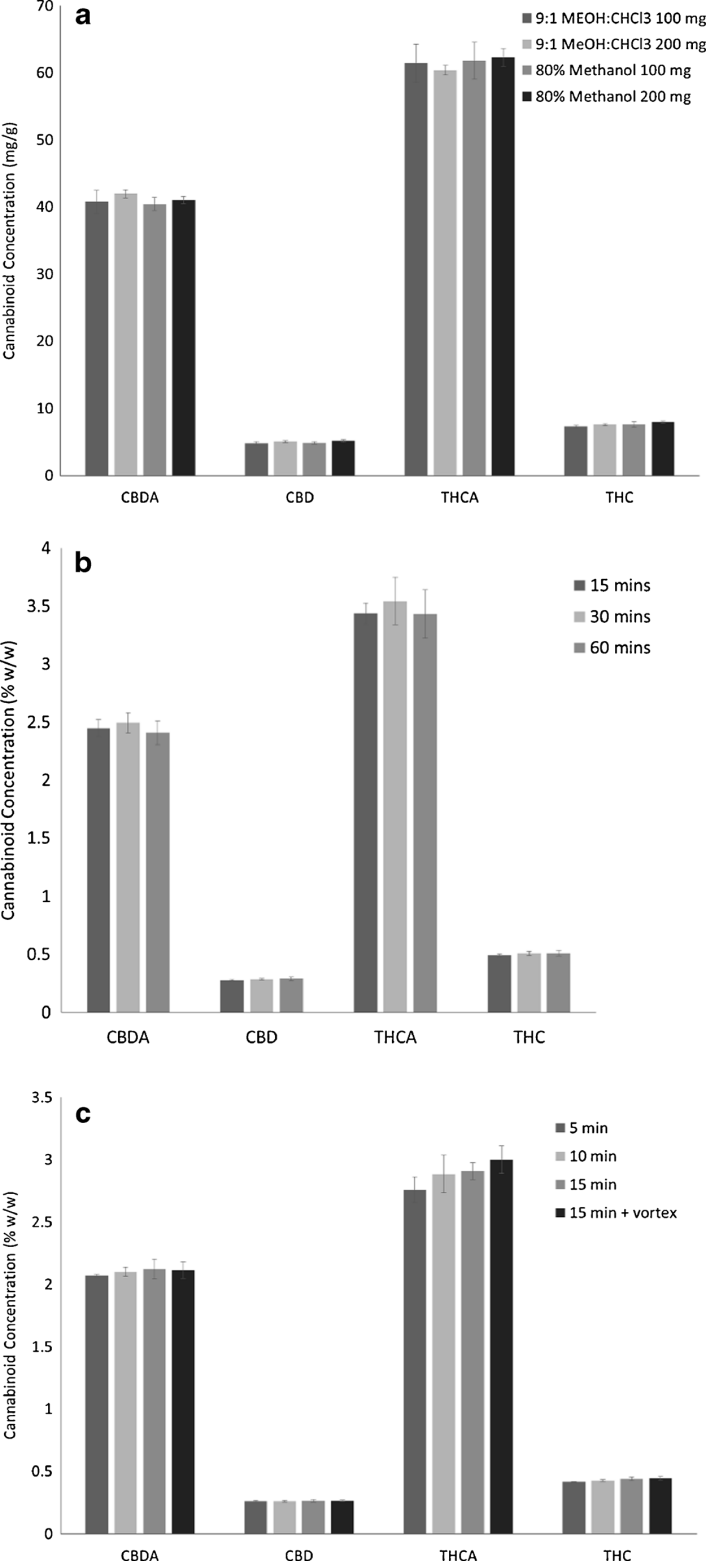
Optimization of a green chemistry extraction protocol. Concentration of the four major cannabinoids comparing **a** solvent composition and volume to mass ratio, **b** extraction times of 15 min, 30 min, and 60 min, **c** short extraction times of 5 min, 10 min, 15 min, and 15 min with vortexing every 5 min. (*n* = 3)

Two levels were selected for each factor: solvent composition (80% methanol, 9:1 methanol/chloroform), extraction technique (sonication, wrist action shaking), extraction time (15 min, 1 h), and solvent volume (10 mL, 25 mL). An initial prescreening of solvents indicated that the extraction efficiency of methanol/chloroform mixture was not different from 80% methanol allowing further studies to use the greener option. The statistical analysis of these data indicated that solvent volume was the most significant factor, followed by solvent composition (Fig. 2). Extraction technique and time did not affect the extraction of cannabinoids. Further studies evaluating the solvent volume to mass and solvent composition using 25 mL extraction solvent with 100 and 200 mg of sample confirmed that 200 mg was equivalent to 100 mg, without saturating the extraction solvent (Fig. 3a). The mass to volume ratios used previously range considerably. In some cases, the mass of sample was as high as 100 mg in 1 mL, up to 500 mg in 100 mL [11, 23]. Although extraction time did not significantly impact the resulting cannabinoid content, it was optimized to increase sample throughput. The factorial design showed slightly lower total cannabinoids at 60 min in comparison with 15 min, potentially indicating some degradation during long extractions. Three time points were assessed: 15, 30, and 60 min. The level of cannabinoids was not significantly different between the time points (Fig. 3b). It was then verified if extraction time could be reduced by evaluating 5, 10, and 15 min in comparison with 15 min with vortexing every 5 min. Again, no significant differences were observed between all four extraction times, while 15 min with vortexing was significantly higher than 5 min (Fig. 3c). These data were used to formulate an optimized standard operating protocol (see Electronic Supplementary Material, ESM) using 200 mg of dried flowers with 25 mL of 80% aqueous methanol for 15 min by sonication with vortexing every 5 min.

#### Extraction of oils

The extraction method was also optimized for cannabis oil comparing (a) 9:1 methanol/chloroform as used in the UNODC method for cannabis products [24], (b) 80% methanol, and (c) 100% methanol. These data show that 80% methanol was not sufficient for extracting the cannabinoids in an oil matrix, but 100% methanol was equivalent to the methanol/chloroform mixture. The final optimized standard operating protocol (see ESM) for cannabis oil samples was 50 mg of oil extracted with 10 mL of methanol using the same extraction parameters as the dried flowers.

#### Stability

The stability of cannabinoids was assessed to determine whether losses occur prior to analysis that may significantly affect the quantitative data. Mixed calibration standards prepared in 80% methanol were stored at −20 °C, 4 °C, and 22 °C in the dark. Variations of less than 5% were considered stable and acceptable. Significant changes in the cannabinoids were found after 30 h at room temperature with THCA/CBDA contents decreasing by 6.3 and 9.6%, respectively, while mixed standards stored at −20 °C were degraded after 48 h with THCA and CBDA contents reduced by 8.1 and 10.6%, respectively. The standards were stable at 4 °C for the duration of the 72-h study. Sample extracts were prepared with 80% methanol and stored at 22 °C in light and dark conditions. Under these conditions, THCA and CBDA were considered unstable by 24 h into the study, with reductions of 6.7% for both, resulting in 8–10% increases in the neutral forms of these cannabinoids. Reductions of 11–23% of acidic cannabinoids occurred under light conditions within 24 h. On the basis of these findings an additional study was performed to evaluate extract stability in 80% methanol and 9:1 methanol/chloroform stored in the dark at 22 °C and 4 °C. The 9:1 methanol/chloroform extracts were found to be stable at 22 °C and 4 °C for the duration of the study, 36 and 48 h, respectively, while 80% methanol at 4 ° C was stable for 48 h. The extracts in 80% methanol were not stable at room temperature for 24 h, similar to the previous study. The elimination of chloroform from the extraction solvent improves analyst safety, reduces solvent costs, and maintains cannabinoids contents; therefore 80% methanol is a viable alternative extraction solvent for analytical quantitative analysis with the use of a dark, temperature-controlled autosampler.

#### Optimized chromatography

**Fig. 4:**
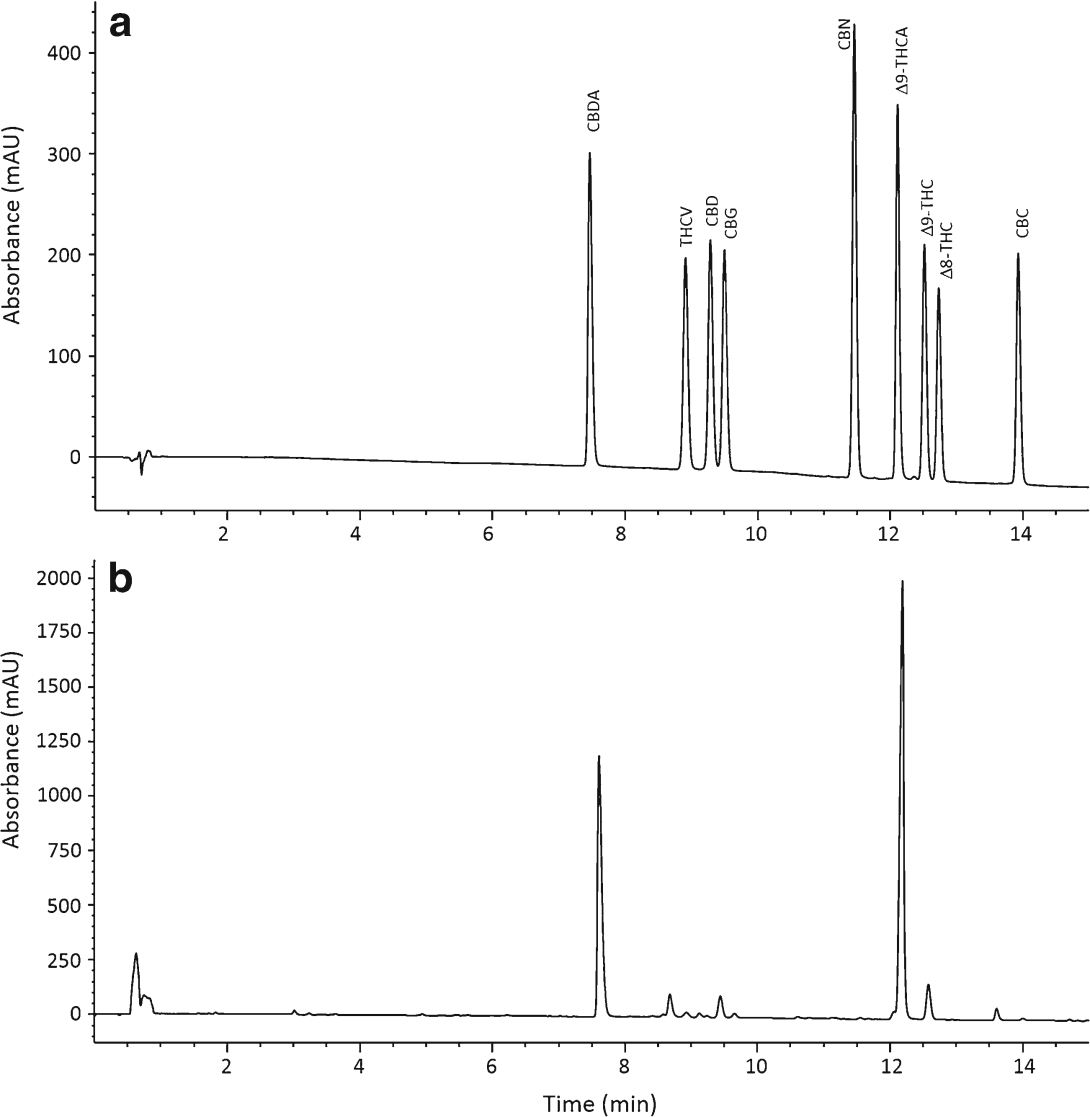
HPLC separation of **a** standard mixture of cannabinoid standards and **b** *Cannabis* flower extract at 220 nm

Several HPLC columns, phases, dimensions, mobile phases, and gradients were compared in preliminary experiments to determine a method for baseline separation of as many cannabinoids as possible while maintaining a short separation time. The optimal column used for separation of cannabinoids was a Phenomenex Kinetex core shell C18 column with sub-2.0-micron particle size. This required an HPLC system capable of pumping above 400 bar, but a 600-bar instrument was sufficient. Mobile phase pH appeared to be a significant factor in cannabinoid separation; as pH decreases, retention of cannabinolic acids increases, while at higher pH the retention decreases with poor peak shape. By optimizing the pH of the aqueous mobile phase for the separation to 3.6, it was possible to separate THCA from other cannabinoids, while maintaining adequate peak shapes. The final methodology was a 15-min analytical run time with 10 mM ammonium formate pH 3.6 and acetonitrile as the mobile phase as shown in Fig. 4. Resolutions of major cannabinoids were greater than 2.0, while minor components were greater than 1.70 using the mixed calibration standards. Sample extracts were used to confirm resolution during the validation study.

### Method validation

#### Linearity

The seven-point calibration curves used on each day of the validation were linear on visual inspection. The correlation coefficient (*r* ^2^) for each cannabinoid was greater than 99.5% for all calibration curves on each day of the analysis, as summarized in Table 1. The plots of residuals were random, confirming that linear functions were suitable for cannabinoid concentrations up to 250 μg/mL. Concentrations of CBDA in the high-THC products CAN004 and CAN007 were lower than the lowest concentration standard used in the validation study. In this case, the materials are outside the calibration range of the method. If samples are known to have lower concentrations, the calibration curves can be adjusted to match the materials, therefore allowing for quantitation within the calibration range. In this case, a calibration range from 2.5 to 50 μg/mL would be sufficient for quantitation. One additional standard would be necessary to run in this case, and a lower concentration range curve could be generated independent of the typically employed curve from 5 to 250 μg/mL.

#### Selectivity

The chromatographic profiles of *Cannabis* extracts at 220 nm were used to evaluate peak resolution. The peak resolutions for the eight cannabinoids quantified with this method ranged from 1.64 to greater than 2.0 as specified in Table 1; in accordance with AOAC guidelines for dietary supplements and natural products, a resolution greater than 1.5 is sufficient for quantitation given the complexity of natural products [20]. Sample extracts were used to evaluate peak purity and confirm resolution with minor peaks. Peak purity was greater than 99% for all cannabinoids evaluated in this assay.

#### Repeatability

Repeatability was assessed by quantifying the eight minor and major cannabinoids for which standards were available. The quantitative data from the four replicate samples on day 1 were used to determine method repeatability. All precision measurements used authentic *Cannabis* materials with a range of cannabinoid concentrations. The repeatability data are summarized in Table 2. Repeatability RSDs ranged from 0.78 to 10.08%. For materials with higher than 0.5% w/w cannabinoid content, the % RSDs ranged from 0.78 to 7.64%; only two of these materials had RSDs greater than 5%. Given that the precision for seven of the nine materials evaluated was less than 5% for the analytes above 0.5% w/w, it is possible that the results for the two materials with greater than 5% RSDs are due to inherent variability of the cannabinoids within these two test samples, rather than an indication of method performance. This is likely due to a higher inherent variability compared with the other strains used in the study. The RSDs over 5% for all other materials were observed for low level cannabinoids, for which small variations in the quantitative data will have more of an impact on the % RSDs. These values are within acceptable validation limits based on their concentrations [20].

**Table 2:** Repeatability and intermediate precision for cannabinoid quantitation in *Cannabis* dried flowers

Sample ID	Cannabinoid	Concentration (% w/w)	Repeatability (% RSD)	Intermediate precision (% RSD)	HorRat
*Cannabis* flowers *ID # CAN001*	CBDA	3.61	2.74	4.26	1.3
	CBD	0.22	3.82	6.01	1.2
	CBG	0.07	6.19	7.13	1.2
	CBN	0.02	8.45	9.92	1.4
	THCA	7.81	3.40	2.85	1.3
	THC	0.67	3.32	4.44	1.1
	CBC	0.03	6.12	11.41	1.7
*Cannabis* flowers *ID # CAN002*	CBDA	3.70	2.86	3.78	1.2
	CBD	0.49	2.68	2.96	0.7
	CBG	0.02	10.08	10.08	1.4
	THCA	0.11	3.29	3.17	0.6
	THC	0.03	3.56	11.61	1.7
	CBC	0.04	2.17	2.97	0.5
*Cannabis* flowers *ID # CAN003*	CBDA	5.97	2.35	3.32	1.0
	CBD	0.25	2.05	4.75	1.0
	CBG	0.10	1.51	3.96	0.7
	CBN	0.02	3.90	7.77	1.1
	THCA	10.3	1.80	3.65	1.3
	THC	0.84	1.75	3.25	0.8
	CBC	0.03	4.49	7.40	1.1
*Cannabis* flowers *ID # CAN004*	CBDA	0.04	2.97	11.13	1.7
	CBG	0.12	3.78	4.02	0.7
	CBN	0.05	3.17	8.06	1.4
	THCA	13.9	2.62	3.50	1.3
	THC	1.74	2.39	3.21	0.9
	CBC	0.06	3.63	6.37	1.0
*Cannabis* flowers *ID # CAN005*	CBDA	7.81	1.76	2.69	0.9
	CBD	1.43	1.51	2.08	0.6
	CBG	0.06	1.97	4.92	0.8
	CBN	0.06	4.29	3.96	0.7
	THCA	2.90	1.84	2.96	0.9
	THC	0.95	2.03	3.20	0.8
	CBC	0.09	3.57	3.53	0.6
*Cannabis* flowers *ID # CAN006*	CBDA	6.27	1.61	4.09	1.4
	CBD	0.70	1.08	3.48	0.8
	CBG	0.11	1.06	5.78	1.0
	CBN	0.04	4.94	4.34	0.7
	THCA	8.67	0.78	5.64	2.0
	THC	1.06	1.35	3.75	1.0
	CBC	0.05	3.50	3.73	0.6
*Cannabis* flowers *ID # CAN007*	CBDA	0.04	4.29	11.67	1.8
	THCV	0.03	4.26	5.24	0.8
	CBG	0.24	4.77	5.06	1.0
	CBN	0.15	2.77	3.43	0.7
	THCA	14.9	2.72	4.66	1.8
	THC	3.31	3.64	4.01	1.2
	CBC	0.05	7.72	7.80	1.3
*Cannabis* flowers *ID # CAN008*	CBDA	8.14	5.84	5.16	1.8
	CBD	0.56	4.47	5.28	1.2
	CBG	0.07	6.85	7.00	1.2
	CBN	0.03	7.64	6.65	1.0
	THCA	5.39	6.18	5.36	1.7
	THC	0.88	4.53	5.57	1.4
	CBC	0.05	4.20	5.44	0.9
*Cannabis* flowers *ID # CAN009*	CBDA	7.95	7.24	5.94	2.0
	CBD	0.36	4.74	5.11	1.1
	CBG	0.06	4.51	6.28	1.0
	CBN	0.06	3.29	10.87	1.3
	THCA	2.05	7.64	6.65	1.9
	THC	0.27	4.43	3.77	0.8
	CBC	0.03	2.28	4.47	0.7

*Cannabinoids below LOQ are not reported

#### Intermediate precision

The quantitative data from the four replicates on the 3 days of analysis were used to calculate the within-day, between-day, and total standard deviations to determine the intermediate precision of the method. Intermediate precision ranged from 2.07 to 11.67% RSD, as summarized in Table 2. The HorRat ratios used to determine the acceptability of the % RSDs based on concentration ranged from 0.5 to 2.0, which is the acceptable range as specified by AOAC International guidelines [20]. The HorRat ratios ranged from 0.3 to 0.7 for the oils (Table 3), indicating that there is improved precision with homogeneous materials where the cannabinoids are not bound to the plant material. The minor cannabinoid concentrations in the oils were below the quantitation and detection limits; therefore only data pertaining to the major cannabinoids was obtained.

**Table 3:** Repeatability and intermediate precision for cannabinoids in *Cannabis* oil

Sample ID	Cannabinoid	Concentration (mg g^−1^)	Repeatability (% RSD)	Intermediate precision (% RSD)	HorRat
*Cannabis* oil *ID # CANOIL*	CBDA	4.95	0.5	3.1	0.7
	CBD	0.24	1.2	2.0	0.3
	THCA	8.28	0.9	2.7	0.7
	THC	0.66	0.9	4.3	0.7

*Cannabinoids below LOQ are not reported

#### Recovery

The cannabinoid recovery was evaluated for the following four major cannabinoids: CBDA, THCA, CBD, and THC. Three concentration levels were evaluated to represent a high, medium, and low concentration material with stinging nettle as the matrix blank. Recoveries are summarized in Table 4 and are within the acceptable ranges as specified by AOAC guidelines [20].

**Table 4:** Recovery results for the quantitation of major cannabinoids using stinging nettle as the matrix blank (*n* = 3)

	CBDA		CBD		THCA		THC	
Sample ID	Conc (% w/w)	Recovery (%)	Conc (% w/w)	Recovery (%)	Conc (% w/w)	Recovery (%)	Conc (% w/w)	Recovery (%)
High	3.5	97.7	1.0	91.3	3.5	96.1	1.0	90.7
Medium	0.4	92.6	0.4	95.5	0.4	90.7	0.4	96.2
Low	0.1	95.4	0.1	95.3	0.1	97.3	0.1	99.2

#### Limits of detection and quantitation

The method detection limit (MDL) and limit of quantitation (LOQ) were determined using the EPA’s method detection limit procedure [22]. A test sample extract was diluted to very low concentrations to account for issues with closely eluting compounds, which will make detection more difficult for cannabinoids with closely eluting compounds. The detection and quantitation limits for each cannabinoid are summarized in Table 5. The limits for CBD are much higher in comparison with the other cannabinoids because of the number of close eluting peaks in the chromatogram. Most other cannabinoids have sufficient resolution from other unknown peaks which do not impact their quantitation and detection.

**Table 5:** Method detection limit and limit of quantitation of cannabinoids in solution and their respective concentrations in dried flowers using the EPA MDL procedures

Cannabinoid	MDL		LOQ	
	Conc (ppm)	Amt in sample (% w/w)	Conc (ppm)	Amt in sample (% w/w)
CBDA	0.17	0.01	0.47	0.03
THCV	0.19	0.01	0.53	0.03
CBD	1.01	0.06	2.74	0.17
CBG	0.31	0.02	0.84	0.05
CBN	0.16	0.01	0.43	0.03
THCA	0.26	0.02	0.69	0.04
THC	0.09	0.01	0.25	0.02
CBC	0.34	0.02	0.93	0.06

## Discussion

With the rapid expansion of labs analyzing *Cannabis*, it is essential to have robust, versatile analytical methods. The currently available methods have several limitations. For example, the resolution of the minor cannabinoids in some cases has been achieved only by selecting a less sensitive UV wavelength to achieve baseline, while this reduction in sensitivity would impact the quantitation of low level compounds found in many products [9]. Some methods fail to provide sufficient method development information to explain extraction solvent selection, extraction times, potential losses, degradation, or inefficiencies [9, 14, 15]. Many methods use chlorinated solvents with potential negative health and environmental impacts. Other methods use high pH mobile phases pH >5.0 which is above the p*K*_a_ of the cannabinolic acids [14]. We found that this elution system caused the peaks to tail significantly and early eluting peaks were asymmetrical.

The demand for cost-efficient quantitative methods for cannabinoids is growing. Many laboratories engaged in this work lack the expertise for advanced analytical instrumentation, such as mass spectrometric detectors. In this case, these detectors would impose a significant cost increase in infrastructure and expertise. Mass spectrometry (MS) would allow for improved detection limits, selectivity, and sensitivity, but given the performance of this method with UV absorbance detection and the high concentrations of the major cannabinoids, the use of MS detection does not improve the method fitness. Care was taken to ensure that the mobile phases used for this method are mass spectrometry compatible for those who with the instrument capabilities require improved sensitivity and putative identification of additional minor cannabinoids.

Our method is a significant improvement over previous methods that can be used in a variety of settings and has the potential to be expanded for inclusion of new cannabinoids as required. To date, many jurisdictions only require the quantity of total THC and total CBD in the products [25, 26], while with improvements in analytical instrumentation, columns, and detection techniques, the ability to expand the regulations to acids, neutral forms, and minor cannabinoids is straightforward. There is a significant amount of concern around the use of GC for quantitation of cannabinoids using in-injector decarboxylation because of conversion issues, which can reduce the accuracy and precision [24]. This issue is no longer a concern when quantifying cannabinolic acids separately from neutral cannabinoids. The information on acid content is also important for those that do not smoke *Cannabis* as the pharmacology of acids varies compared to neutral cannabinoids [27]. Understanding the cannabinoid profiles of different *Cannabis* strains will allow additional information for clinical researchers to understand the complex composition of these plants and their roles in pain regulation and treatment of a variety of other illnesses.

## Conclusions

We developed an optimized HPLC-DAD method with reduced extraction time and greener solvents for adoption into cannabis testing laboratories. Sample turnaround is significantly reduced, while method validation confirmed that the method produces repeatable, accurate results. The sample preparation eliminates the use of chloroform, which has been routinely used in cannabinoid analysis, reducing material costs, use of greener solvents, and improved laboratory safety for personnel. This method can be used in a variety of settings from clinical studies, research, quality control, and regulatory evaluation of this growing industry.

## Electronic supplementary material

Below is the link to the electronic supplementary material.

ESM 1 (PDF 390 kb)
