# Supplementary material for: Leaner and greener analysis of cannabinoids
Source: Anal Bioanal Chem. 2017 Feb 23;409(12):3153–63. doi: 10.1007/s00216-017-0256-3 (PMC5395585; doi:10.1007/s00216-017-0256-3)
Supplement: Supplementary file 1 — (PDF 390 kb) [file 216_2017_256_MOESM1_ESM.pdf]

Analytical and Bioanalytical Chemistry

Electronic Supplementary Material

Leaner and Greener Analysis of Cannabinoids

Elizabeth M. Mudge, Susan J. Murch, Paula N. Brown

1. SCOPE & APPLICABILITY

This method is to be used to quantify the four major cannabinoids and four minor cannabinoids in *Cannabis sativa* dried, ground flowers and oils. The four major cannabinoids of interest are THCA, THC, CBDA and CBD.

2. STRUCTURES

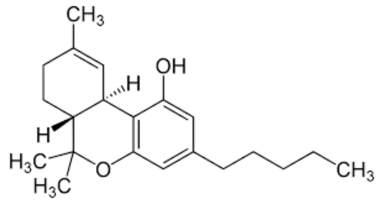

THC

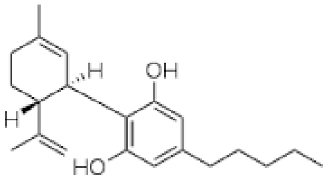

CBD

3. REFERENCE MATERIALS

- Tetrahydrocannabinolic acid (THCA-A), Certified reference standard (1 mg/mL), Cerilliant Corp
- Tetrahydrocannabinol (THC), Certified reference standard (1 mg/mL), Cerilliant Corp
- Cannabidiolc acid (CBDA), Certified reference standard (1 mg/mL), Cerilliant Corp
- Cannabidoil (CBD), Certified reference standard (1 mg/mL), Cerilliant Corp
- Tetrahydrocannabivarin (THCV), Certified reference standard (1 mg/mL), Cerilliant Corp
- Cannabigerol (CBG), Certified reference standard (1 mg/mL), Cerilliant Corp
- Cannabichromene (CBC), Certified reference standard (1 mg/mL), Cerilliant Corp
- Cannabinol (CBN), Certified reference standard (1 mg/mL), Cerilliant Corp

4. REAGENTS

- Water, Nano Pure Deionized
- Methanol, HPLC grade
- Acetonitrile, HPLC grade
- Formic Acid (>99%), HPLC grade
- Ammonium Formate (99%), HPLC grade

5. EQUIPMENT

- Analytical balance
- Coffee grinder
- Volumetric pipette, class A, 25 mL, 10 mL
- Polypropylene conical centrifuge tubes, amber, 50 mL
- Centrifuge, Eppendorf 5804 or equivalent
- Sonicator
- Micropipetters (1000 µL, 200 µL)
- HPLC vials, amber
- HPLC caps
- HPLC: Agilent 1200 equipped with binary pump operating at 600 bar (must be above 400 bar), temperature controlled column, temperature controlled autosampler and UV-Vis detector.
- Column: Phenomenex Kinetex C18 3.0 x 100 mm, 1.7 µm with guard cartridge
- Disposable luer-lok syringes, 3 mL
- Syringe filters, 0.22 µM PTFE
- HPLC solvent bottles, 1 L
- Vortex mixer
- 2 mL micrcentrifuge tubes, amber
- Glass Pasteur pipettes and bulbs

6. PREPARATION OF SOLUTIONS

Extraction solvent (tissues): 80% methanol

Using a graduated cylinder, combine 800 mL of methanol and 200 mL of ultrapure water. Mix thoroughly.

Extraction solvent (oil): 100% methanol

Mobile phase A: 10 mM ammonium formate, pH 3.6

To prepare 1 L of mobile phase, accurately weigh 0.631 grams of ammonium formate onto a weight boat. Add to 1.0 L of ultrapure water (or HPLC grade water). Using a pH meter, adjust the pH of the solution using formic acid (you may need to dilute the formic acid in water prior to adding to the large solution) to pH 3.60. Filter the solution using a 0.2 µm nylon filter. Label bottle and install on HPLC.

Mobile Phase B: Acetonitrile

Filter 1.0 L of HPLC grade acetonitrile using a 0.2 µm nylon filter. Label bottle and install on HPLC.

7. PREPARATION OF CALIBRATION STANDARDS

1000 ppm Stock Solutions

Mixed standards are stable for up to 3 days at 4 °C. In-house verification should be performed to confirm stability.

Prepare working stock solutions for the cannabinoids according to the following table. These will be used to make the mixed calibration standards.

Cannabinoid	Dilution 1	Dilution 2
THCA	n/a	
CBDA		
THC	1:5	n/a
CBD	1:5	
CBG	1:2	1:10
CBN	1:2	1:10
CBC	1:2	1:10
THCV	1:2	1:10

Calibration Standards

Prepare mixed standards as shown in the tables below. Label them as linearity (Lin) 1 through 7. Lin 1-4 are prepared as separate mixed standards. Lin 5-7 are dilutions of Lin 1, 3 and 4, respectively.

Cannabinois	Approximate concentration (µg/mL)						
	Lin 1	Lin 2	Lin 3	Lin 4	Lin 5	Lin 6	Lin 7
THCA	250	200	100	50	25	10	5
CBDA	250	200	100	50	25	10	5
THC	50	40	20	10	5	2	1
CBD	50	40	20	10	5	2	1
CBN	25	20	10	5	2.5	1	0.5
CBC	25	20	10	5	2.5	1	0.5
CBG	25	20	10	5	2.5	1	0.5
THCV	25	20	10	5	2.5	1	0.5

Cannabinoid		Lin-1	Lin-2	Lin-3	Lin-4	Lin-5	Lin-6	Lin-7
THCA/ CBDA	Dilution	n/a	n/a	n/a	n/a	Lin 1 100 µL	Lin 3 100 µL	Lin 4 100 µL
	µL	250	200	100	50			
THC/ CBD	Dilution	n/a	n/a	1:5	1:5			
	µL	50	40	100	50			
CBN/ CBC/ CBG/ THCV	Dilution	1:2	1:2	1:10	1:10			
	µL	50	40	100	50			
80% MeOH		200	360	200	600	900	900	900

8. PREPARATION OF TEST SAMPLES

8.1. Dried Tissues (whole or milled)

1. Grind tissues in a coffee grinder until a fine powder is obtained. Care should be made to reduce the amount of heat buildup during the grinding process.
2. Accurately weigh 200 mg of the sample into a 50-ml amber centrifuge tube.
3. Add 25 mL of 80% methanol with a 25-mL pipette to each tube and vortex for 30 seconds to mix.
4. Extract using the sonicating bath for 15 min at room temperature, vortexing the samples every 5 minutes.
5. Centrifuge at 5000 rpm for 5 minutes.
6. Filter sample through a 0.22 µm PTFE filter into amber 2 mL microcentrifuge tube.
7. Dilute the filtered sample into an amber HPLC vial with 80% methanol. Typical dilutions are 1:5 or 1:10, sometimes 1:2 is acceptable.
8. Analyze for cannabinoid content using the HPLC method described below.

8.2. Cannabis oil

1. Mix the oil by inversion to ensure homogeneous solution.
2. Using a glass Pasteur pipette, pipette 50 mg ±5 mg oil into an amber 50 mL centrifuge tube. (3-4 drops, approx.)
3. Add 10 mL of methanol with a 10-mL pipette to the tube and vortex for 30 seconds to mix.
4. Extract in a sonicating bath for 15 minutes, vortexing every 5 minutes.
5. Centrifuge at 5000 rpm for 5 minutes.
6. Filter sample through 0.2 µm PTFE filter into an HPLC vial.
7. Analyze for cannabinoid content using the HPLC method described below.

9. CHROMATOGRAPHY

9.1. Instrument

A suitable HPLC system equipped with at least:

1) a UV detector capable of monitoring at 220 nm
2) a temperature controlled autosampler
3) a pump capable of delivering constant flow up to 600 bar (must be more than 400 bar) and
4) a computing data processor

9.2. Column

Phenomenex Kinetex C18 100 mm x 3.0 mm, 1.7 µm

9.3. Mobile Phase

Mobile Phase "A": 10 mM ammonium formate, pH 3.6, 0.22 µm filtered
Mobile Phase "B": Acetonitrile, 0.22 µm filtered

9.4. Gradient Program

The mobile phase is changed linearly according to the following program:

Time (min)	% MP B	Flow rate (mL/min)
0	52	0.6
8.0	66	0.6
8.5	70	0.6
13	80	0.6
15	80	0.6

Post run: 7 minutes for column re-equilibration

9.5. Flow Rate

0.6 mL/minute

9.6. Injection Volume

5 µL

9.7. Detection

220 nm

9.8. Run Time

Stop Time: 15 min + 7 minutes post run (22 minutes)

9.9. Column Temperature

25°C

9.10. Autosampler Temperature

4°C

10. PROCEDURE

10.1. Equilibrate the HPLC system with the mobile phases.

10.2. Inject the standards used to produce the 7-point calibration curve.

10.3. Inject the samples

10.4. After every 10 injections, re-inject one of the standards for quality control purposes.

10.5. At the end of the run, re-inject one of the standards for quality control purposes.

11. CALCULATIONS

Integrate the peaks that represent CBDA, THCV, CBD, CBG, CBN, THCA, THC and CBC in the lowest level standard used. This is the elution order of the cannabinoids.

Repeat this for all standard levels used.

Construct a plot of concentration µg/ml of analyte (x-axis) versus the individual peak area abundance (y-axis) for each cannabinoid.

Use a least squares analysis to calculate the slope (β), intercept (α) and correlation coefficient (r^2) of the best-fit line for each curve.

Determine the peaks that correspond to the eight cannabinoids in each sample.

Integrate the identified peaks.

The concentration of each cannabinoid in the sample vial is calculated by the following equation:

$$\text{Cannabinoid Concentration}\left(\frac{\mu g}{mL}\right) = \frac{\text{curcuminoid area} - \text{intercept of linear regression}}{\text{slope of linear regression}}$$

The amount of each Cannabinoid in the original sample is given by the following equation:

$$Cannabinoid\ in\ original\ sample\ (\%w/w) = \frac{Concentration\ (\mu g/mL) \times Volume(mL)}{Mass\ (mg)} \times 10 \times D$$

where: **C**= Concentration of analyte (mg/L) from linear regression analysis

V = Initial volume (mL)
W = mass of sample (mg)
D = dilution factor
10 = conversion from mg/g to %w/w

Repeat the calculations for all replicates and samples. Calculate the mean and standard error for each of the sample sets. Report the amount of alkaloids in each sample set utilizing a 95% confidence as per the following formula:

$$\text{Reported Amount} = \overline{X} \pm t_{\alpha,v} \cdot s_{\bar{x}}$$

where: $\overline{X}$ = Mean of sample set

$t_{\alpha,v}$ = t critical value.

For samples of 3 replicates (i.e. υ = 2) and α = 0.05, critical value = 4.303
For samples of 4 replicates (i.e. υ = 3) and α = 0.05, critical value = 3.182

$s_{\bar{x}}$ = Size of standard error

The size of the standard error = $s/\sqrt{n}$

where: s = standard deviation
n = number of replicates

Note: it is recommended that at least 3 replicates are performed for each sample.

12. TYPICAL CHROMATOGRAMS

Below are typical chromatograms obtained using HPLC-DAD scanning at 220 nm for a standard mixture sample and a cannabis flower and oil sample.

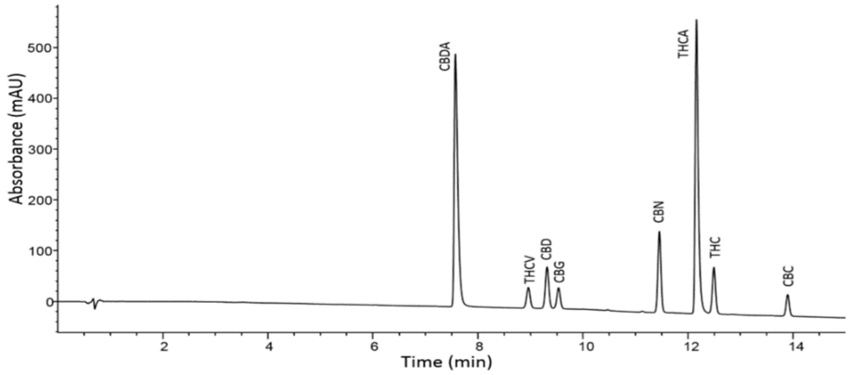

Fig. S1 Chromatogram of a mixed standard solution containing of all eight cannabinoids analyzed by HPLC-DAD scanning at 220 nm. The peaks corresponding to the cannabinoids are indicated

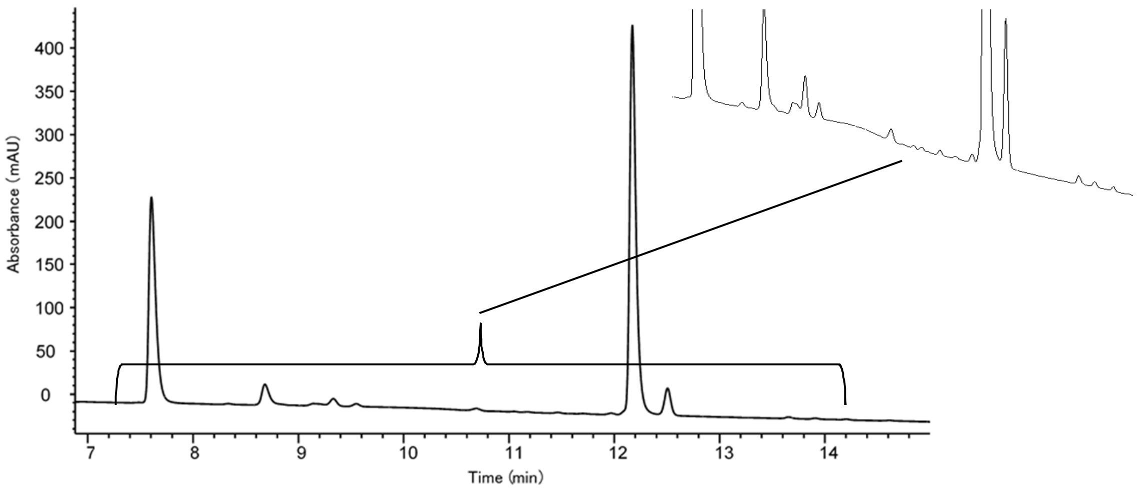

Fig. S2 Typical chromatogram of a cannabis flower sample analyzed by the described analytical method

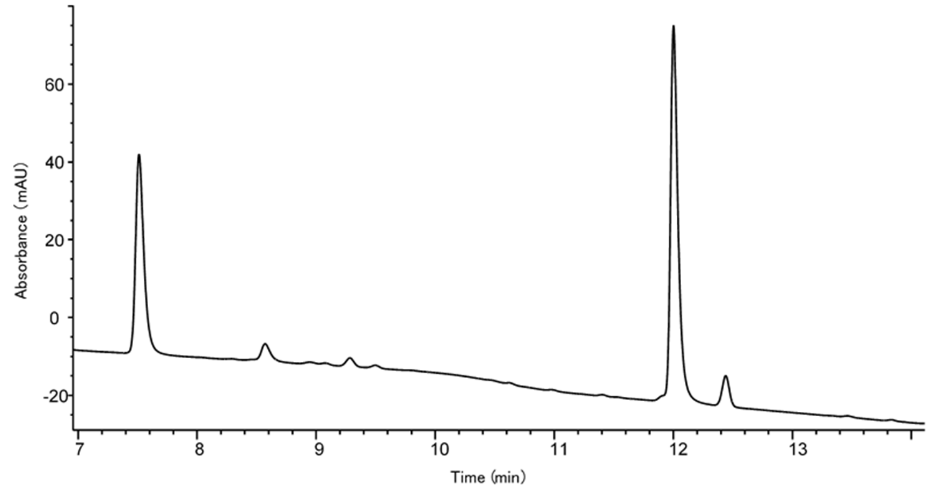

Fig. S3 Typical chromatogram of a cannabis oil sample analyzed by the described analytical method

13. TYPICAL CALIBRATION CURVES

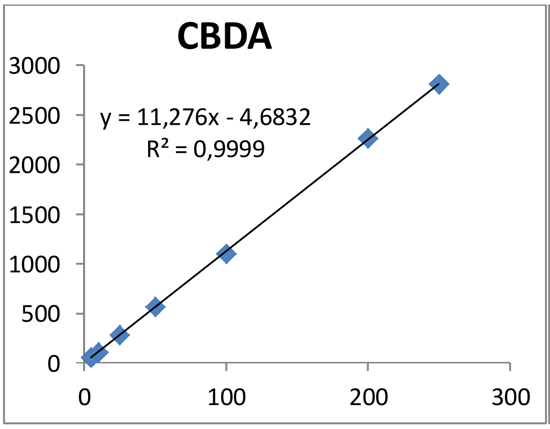

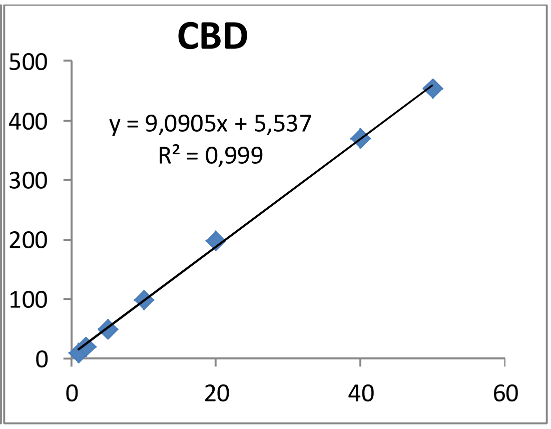

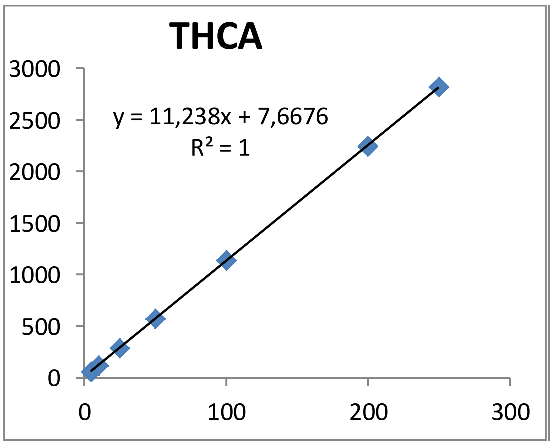

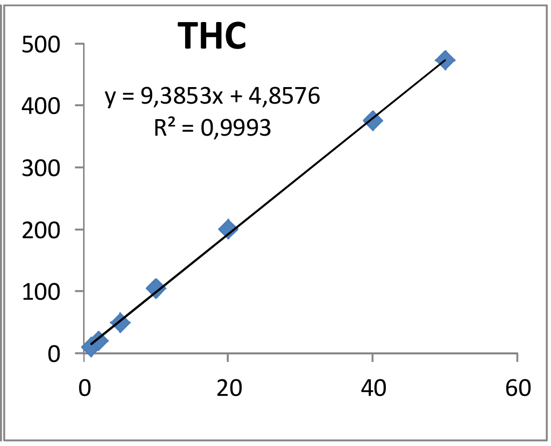

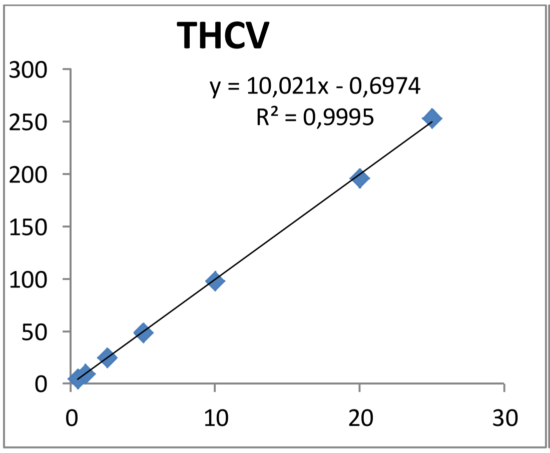

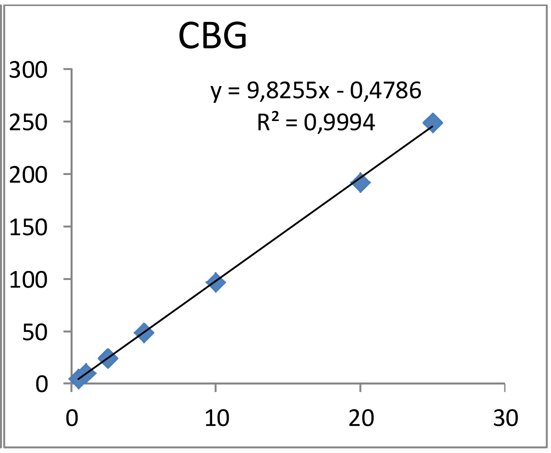

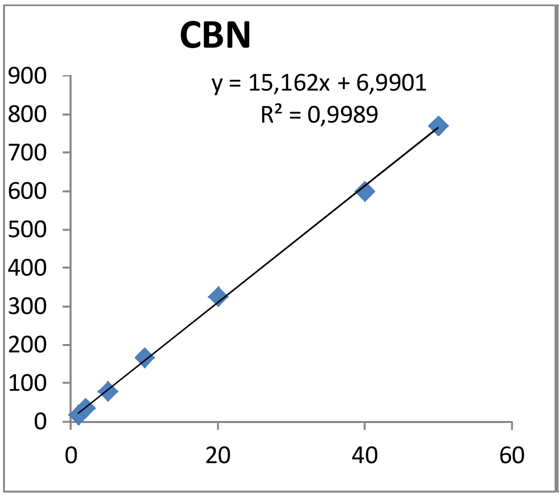
CBN
y = 15,162x + 6,9901
R² = 0,9989
900
800
700
600
500
400
300
200
100
0
0
20
40
60

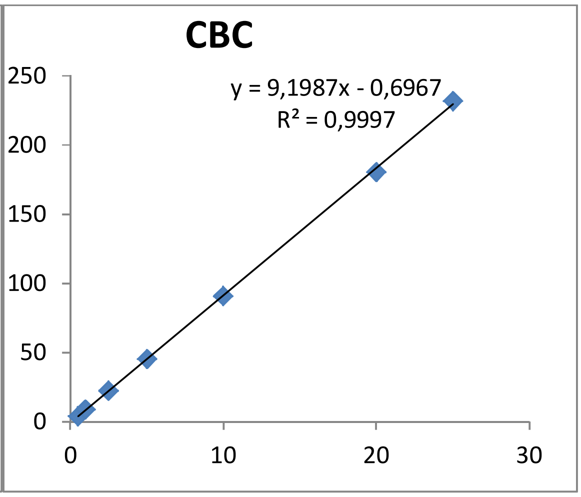
CBC
y = 9,1987x - 0,6967
R² = 0,9997
250
200
150
100
50
0
0
10
20
30
